# Strategies That Utilize Ion Pairing Interactions to Exert Selectivity Control in the Functionalization of C–H Bonds

**DOI:** 10.1021/jacs.2c08752

**Published:** 2022-09-30

**Authors:** James E. Gillespie, Alexander Fanourakis, Robert J. Phipps

**Affiliations:** Yusuf Hamied Department of Chemistry, University of Cambridge, Lensfield Road, Cambridge CB2 1EW, U.K.

## Abstract

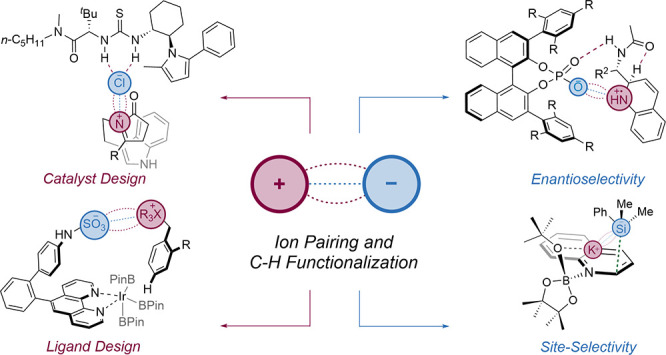

Electrostatic attraction between two groups of opposite charge, typically known as ion-pairing, offers unique opportunities for the design of systems to enable selectivity control in chemical reactions. Catalysis using noncovalent interactions is an established and vibrant research area, but it is noticeable that hydrogen bonding interactions are still the main interaction of choice in system design. Opposite charges experience the powerful force of Coulombic attraction and have the ability to exert fundamental influence on the outcome of reactions that involve charged reagents, intermediates or catalysts. In this Perspective, we will examine how ion-pairing interactions have been used to control selectivity in C–H bond functionalization processes. This broad class of reactions provides an interesting and thought-provoking lens through which to examine the application of ion-pairing design strategies because it is one that encompasses great mechanistic diversity, poses significant selectivity challenges, and perhaps most importantly is of immense interest to synthetic chemists in both industry and academia. We survey reactions that proceed via radical and ionic mechanisms alongside those that involve transition metal catalysis and will deal with control of site-selectivity and enantioselectivity. We anticipate that as this emerging area develops, it will become an ever-more important design strategy for selectivity control.

## 1 Introduction

Ionic interactions, often referred to as ion-pairing interactions, are one of the most fundamentally important noncovalent interactions and result from electrostatic attraction between two groups of opposite charge. The strength of an ionic interaction is dictated by Coulomb’s law, in which the attractive force is a function of the distance between the charges and the dielectric constant ε of the medium. Gas phase (ε = 1) attraction between a simple cation and anion can result in binding energies measured in the hundreds of kcal/mol although solvent effects can severely curtail this, with minimal interaction between ions in water (ε = 78).^1^ Chemical reactions are often carried out in solvents that possess dielectric constants far closer to the gas phase, such as dioxane (ε = 2), toluene (ε = 2), and chloroform (ε = 5). Coulomb’s law calculates that two opposite point charges 5 Å apart in 1,4-dioxane would experience an attraction energy of approximately 30 kcal/mol.^2^ This is clearly a significant magnitude of energy and is able to occur at a range beyond which other noncovalent interactions such as hydrogen bonding can typically operate.

The fundamental roles played by noncovalent interactions in biological systems have long been appreciated, and the establishment of supramolecular chemistry thrust them to center-stage as key components in molecular assembly, resulting in detailed study and quantification.^3^ Noncovalent interactions have increasingly been recognized as powerful design tools for methods development, enabling substrates to engage with reagents or catalysts. This can at the least allow proximity effects to increase reaction rates but, more excitingly, can be exploited to exert control over reaction selectivity. Noncovalent organocatalysis has emerged as a distinct field over the past two decades and the incorporation of noncovalent design strategies into small molecule catalysis is now very much in the mainstream.^4^ Attractive noncovalent interactions are also increasingly being incorporated into ligand designs for transition metal complexes to enable selectivity control,^5^ and a number of reviews in recent years have highlighted the breadth of control strategies that have been explored using noncovalent interactions in a general sense.^6^

It is noticeable that in catalyst designs, hydrogen bonding is by far the most widely employed of the suite of available noncovalent interactions.^7^ A good reason for this is the high directionality of hydrogen bonding; this can give confidence that a particular orientation planned “on paper” may hold true in reality, particularly if two hydrogen bonds are used in tandem such as in urea or thiourea catalysis.^7,8^ Another advantage is the fact that many common functional groups can act as hydrogen bond donors or acceptors to some degree, reducing the need for esoteric or bespoke functionality. Given that hydrogen bond strengths are, in general, weaker than ion pairs at most separations, it is somewhat surprising that ion pairs have been explored to such a lesser extent. One likely reason for this is the perceived low directionality of ion-pairing interactions, which may be assumed to preclude precise chemical positioning and therefore impact ability to exert control. While there is undoubtedly truth to this, one could also argue that we still do not yet have the ability to exactly design the perfect system. Furthermore, if such a precise system is required, then it could suffer from limited substrate generality, typically considered a disadvantage in synthetic methodology development. If such a precise level of control is not needed to achieve useful selectivity, then the “flexibility-to-fit” of a looser ion-pairing interaction could mean that one catalyst design might “fit” a number of different substrate classes, rather than having to be redesigned each time. In this sense, ion-pairing could, in principle, offer valuable advantages over hydrogen bonding in terms of generality, as well as strength. It should also be remembered that in many systems weaker, but directional, hydrogen bonds may also be at play in subtle ways. For example, it is well established that C–H bonds adjacent to a quaternary ammonium can act as hydrogen bond donors, giving directionality to the interaction with the associated anion.^9^ Furthermore, many ionic catalysts possess additional functionality, which can engage in either a second interaction with the substrate or a second reaction component, in both cases providing a higher degree of organization.

To consider how broadly applicable ion-pairing strategies could be, a glance at areas in which they have already been explored for selectivity control provides very encouraging indicators. One of the first was the use of chiral cations to exert enantiocontrol in enolate alkylation under phase-transfer conditions, a catalysis mode that first established the viability of ion-pairing for enantioselectivity control in a general and practically useful reaction class.^10^ Since then, chiral cations have been explored more widely including, to a limited extent, in combination with transition metal catalysis.^11^ A distinct mode in which the charge on the chiral catalyst is inverted emerged in the mid-2000s and was largely triggered by the introduction of chiral phosphoric acids as versatile scaffolds for asymmetric catalysis.^12^ “Chiral anion catalysis” or “asymmetric counteranion-directed catalysis’” utilizes chiral anions to pair with cationic reaction components in catalytic cycles.^13^ The relatively common occurrence of cationic metal complexes enabled transition metal catalysis to be used with chiral anions.^14^ Ion-pairing of chiral phosphates with organic cationic intermediates such as iminium ions^15^ and aziridinium ions,^16^ as well as cationic reagents,^17^ has been extensively investigated. Chiral anion-binding exploits ion-pairing in a different manner whereby an achiral anion is bound by a chiral anion-binding catalyst.^13c^ Despite the more elaborate arrangement, ion-pairing is still central to the interaction, which defines stereochemistry in the product, and this has been used to enable a range of highly enantioselective transformations, some of which will be discussed later in this article.^18^

In this Perspective, we will examine how ion-pairing interactions have been applied to exert control of selectivity in a particular category of transformation that encompasses mechanistically diverse chemistry and is also highly desirable to end-users: the functionalization of C–H bonds.^19^ The net replacement of the hydrogen atom in a C–H bond with a different atom or new group is an efficient way to build up molecular complexity, and this broad field of study has grown immensely in the past decades. This general description can cover a wide remit, encompassing numerous different mechanisms. In considering examples to illustrate this Perspective, we will deliberately adopt a fairly broad definition of what constitutes a C–H bond functionalization process, borrowing the definition from Holmberg-Douglas and Nicewicz in their recent review on Photoredox-Catalyzed C–H Functionalization Reactions: “any organic transformation of a C–H into a C–X bond without a change in the oxidation state of the substrate”.^19f^ An exception to this is that we will not include examples of carbonyl α-functionalization, which have been extensively reviewed elsewhere in the context of asymmetric phase-transfer catalysis.^10b,10c^ We choose to only cover examples that invoke ionic interactions between two oppositely charged ions of full charge, referred to as ion-pairing. There are other examples that may invoke electrostatic interactions between polarized areas of molecules or groups, but these will not be covered as we seek to keep the focus on how discrete charges may be used more readily in the explicit design of systems. It should be emphasized that this is not a Review article, and it is not the aim to cover all examples of particular reaction types but rather to give a representative overview of the approach as a whole.

The fact that most molecules possess multiple C–H bonds means that site-selectivity (or regioselectivity, depending on the context) is a very important consideration.^20^ Furthermore, the functionalization of C–H bonds can lead to enantiomers, depending on the reaction type, and rendering such processes enantioselective can be challenging.^21^ This Perspective will explore how ion-pairing design strategies have impacted this important and topical type of chemical reaction. For clarity, it will be divided into three sections based on the mechanism type: radical, ionic, and metal-catalyzed. Within each section, we will discuss site-selectivity and enantioselectivity in turn. Finally, we will provide an outlook considering future challenges and opportunities, which will hopefully inspire other researchers to design systems for selectivity control based on ion-pairing interactions for the control of C–H functionalization and perhaps even other reaction classes in addition.

## 2 Radical Mechanisms

### 2.1 Site-Selectivity

**Figure 1:**
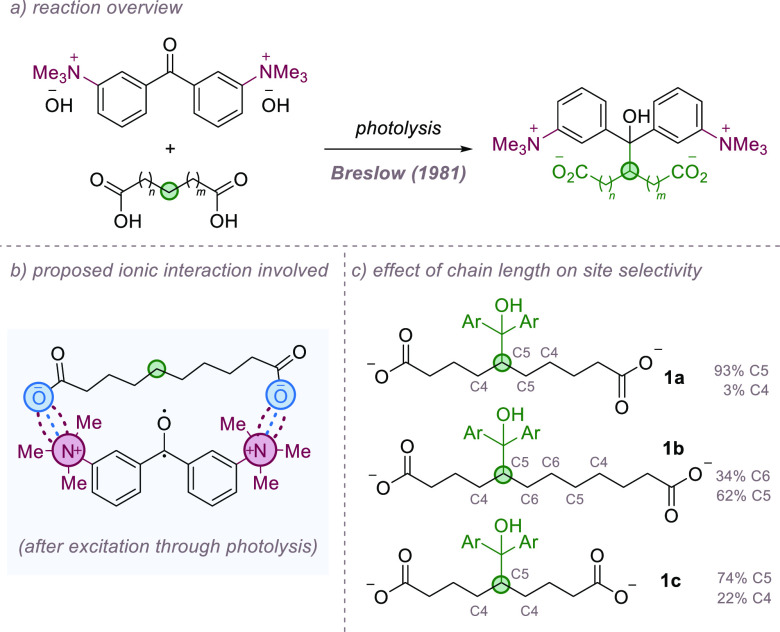
Site-selective functionalization of long chain diacids using dual ion-pairing interactions.

The majority of examples in which ion-pairing interactions have been used to exert control of site-selectivity in radical C–H functionalization reactions have exercised control using hydrogen atom transfer (HAT) as the key step.^19f,22^ Two pioneering examples arose from Breslow and co-workers in the early 1980s. In the first, the authors showed that flexible, long chain diacids could be selectively functionalized by a rigid dicationic benzophenone reagent bearing trimethylammonium groups on the *meta* position of each ring (Figure 1a).^23^ Upon mixing the dicationic benzophenone salt with diacids of various length, an ion-paired complex was proposed to form. The ion-pairing interaction between the benzophenone and diacid was thought to rigidify the reactant conformation, and it was hypothesized that upon photolysis, site-selective C–H abstraction may occur in partners that were well matched (Figure 1b). Decanedioic acid (**1a**) reacted with impressive selectivity, with 93% functionalization occurring at the two equivalent C5 positions (Figure 1c). Tellingly, when the two-methylene longer chain dodecanedioic acid (**1b**) was used, the site-selectivity dropped, with 62% functionalization at the equivalent C5 positions and 34% at C6, thought to be because the dianion is now too long to rigidly ion pair with the benzophenone with its chain fully extended and must therefore kink, impacting selectivity. When shorter nonanedioic acid (**1c**) was used, 74% of functionalization occurred at the single C5 atom with a total of 22% occurring at the neighboring C4 atoms. This was a very astute early example probing the application of ion-pairing to control HAT selectivity and was certainly ahead of its time.

**Figure 2:**
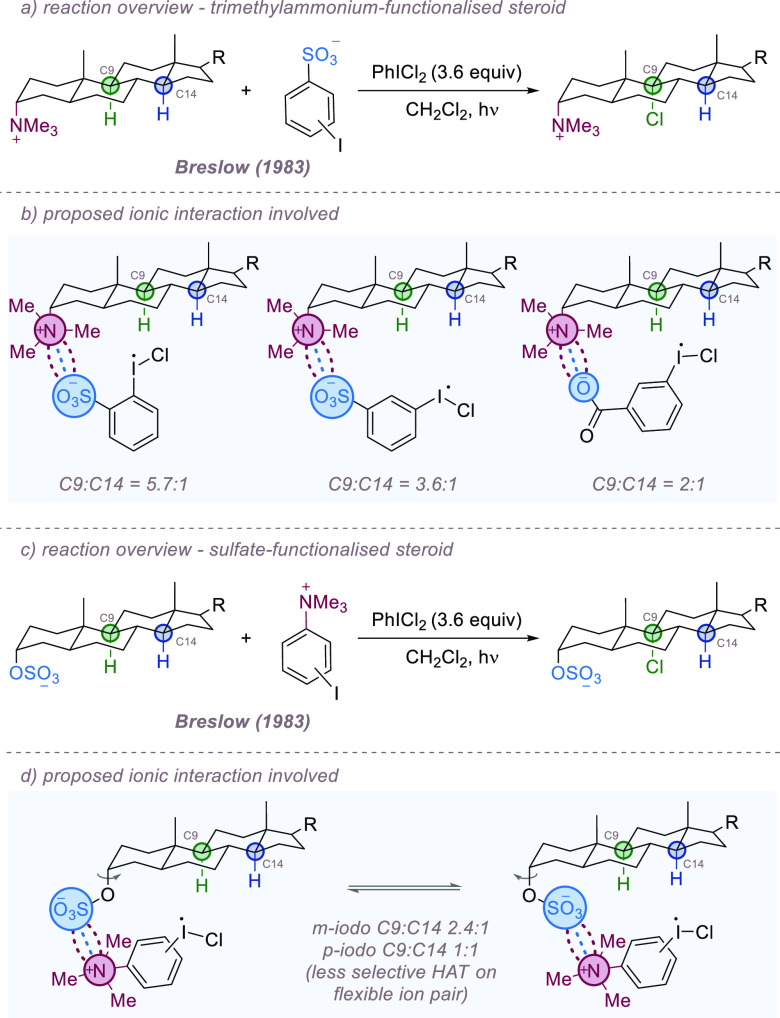
Site-selective chlorination of steroids using an iodoarene associated through ionic interactions.

In the second, Breslow and Heyer examined related ion pair directed C–H functionalization in the context of their laboratory’s extensive steroid functionalization studies.^24^ Charged hypervalent iodine reagents were ion-paired with oppositely charged cholesterol derivatives, and the site-selectivity of subsequent C–H chlorination of the steroid scaffold was probed.^25^ Initially the authors prepared a trimethylammonium cholestanyl cation and partnered it with various aryl iodides rendered anionic by sulfonate or carboxylate substituents on the aromatic ring (Figure 2a). Treatment of these salts with PhICl_2_ for 30 min, followed by irradiation with a sun lamp, resulted in chlorination of C(sp^3^)–H bonds at either of the tertiary C9 or C14 carbons. Site-selectivity was proposed to be determined by the geometrical proximity of the iodine substituent, which would relay a chlorine atom to the steroid framework, resulting in hydrogen atom abstraction. Use of a *meta*-iodinated benzenesulfonate anion gave a 3.6:1 C9/C14 ratio, whereas the *ortho* isomer gave an improved ratio of 5.7:1 (Figure 2b). A *meta*-iodinated benzoate counterion was also evaluated, but this gave reduced selectivity of 2:1. The authors also inverted the charges and rendered the cholesterol derivative anionic by the incorporation of a sulfate group, allowing it to be partnered with various benzenetrimethylammonium cations (Figure 2c). Direct comparison with the previous best system was difficult as the *ortho*-iodinated cation was not tested, but for the *meta* isomer, C9/C14 selectivity was lower, giving a C9/C14 ratio of 2.4:1, and for the *para* isomer, a 1:1 ratio was obtained (Figure 2d). This reduced selectivity is likely due to the increased flexibility of the sulfate group in comparison to the trimethylammonium group initially studied. Crucially, in the absence of a charged group on each component, no reactivity was observed, and the authors noted that this indicates formal catalysis, even if turnover is not achieved. While the efficiencies and selectivities of these early examples were moderate, they showcased the potential for using ion-pairing interactions to exert control over site-selectivity in the functionalization of C–H bonds using radical chemistry.

**Figure 3:**
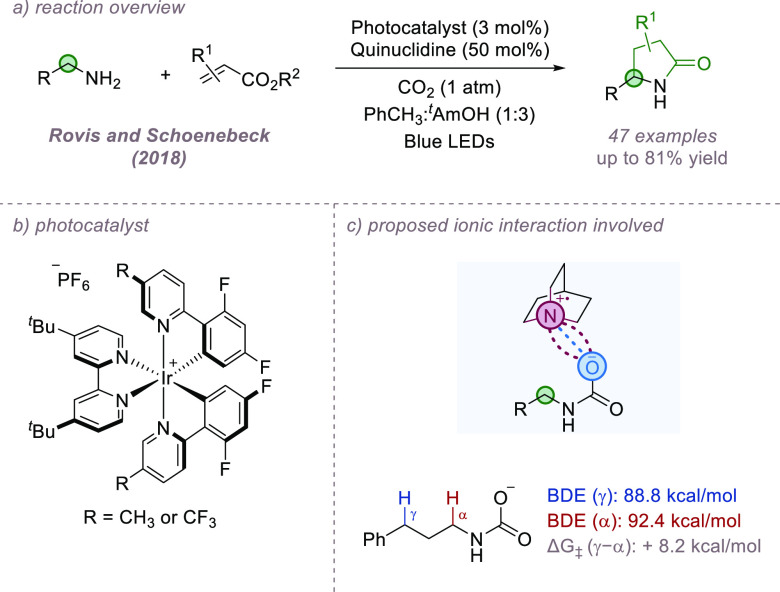
Site-selective alkylation of primary amines in which ion pair directed HAT is implicated.

In 2018, Rovis and Schoenebeck demonstrated the site-selective α-alkylation of primary aliphatic amines using a combination of HAT and photoredox catalysis, allowing efficient synthesis of γ-lactams (Figure 3a,b).^26^ Important to the success of this reaction was an atmosphere of CO_2_, and the authors propose that CO_2_ initially reacts with the amine to form a carbamate anion. This allows ion-pairing to occur between it and a quinuclidinium radical cation, directing site-selective HAT to occur from the position α to the carbamate group. The HAT step occurred with excellent site-selectivity even when other susceptible C–H bonds were present, such as at benzylic and tertiary positions, and those adjacent to heteroatoms. Computational studies suggested that the bond-dissociation energy (BDE) for the benzylic C–H bond was approximately 4 kcal/mol lower than that of the C–H bond α to the anionic carbamate (Figure 3c). However, the free energy barrier for C–H abstraction at the latter position was 8.2 kcal/mol lower due to the stabilizing ion-pairing interactions between the carbamate anion and the quinuclidinium radical cation. Reactivity enhancement linked to the proposed ion-pairing was also observed. Rovis and co-workers subsequently reported a related α-selective alkylation of alkyl triflamides.^27^ The triflamide was thought to be deprotonated under the reaction conditions, making the α-C–H bond more hydridic and susceptible to abstraction by the quinuclidinium radical cation. However, the authors note that an ion-pairing interaction between the triflamide anion and quinuclidinium cation could not be discounted as playing a possible role in site-selectivity.

**Figure 4:**
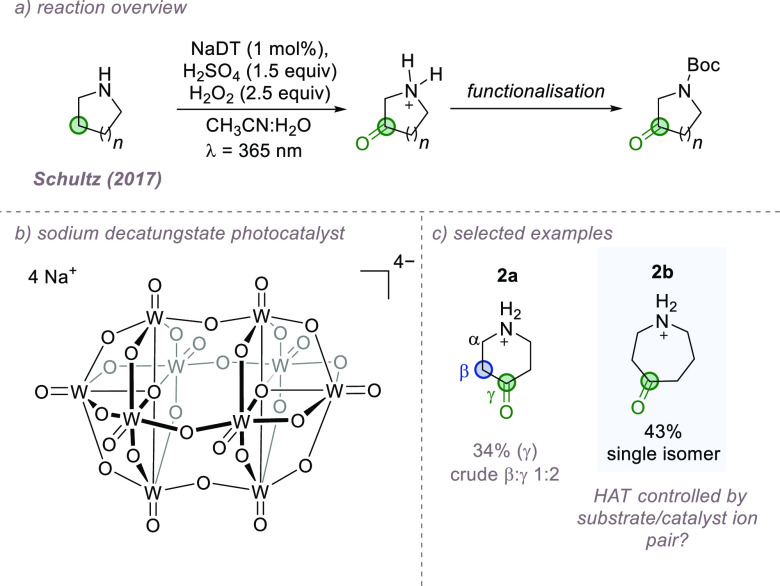
Remote oxidation of amines using NaDT catalysis and the unexpected site-selectivity observed in **2b**.

Polyoxometalate anions are gaining considerable attention as versatile HAT catalysts that are able to abstract stronger alkyl C–H bonds when photoexcited, particularly those not activated by a neighboring heteroatom.^28^ Specifically, the decatungstate anion [W_10_O_32_]^4–^ has been extensively explored and is of particular interest to this discussion due to its charged nature and the potential opportunities for exploiting ion-pairing interactions. In 2017, Schultz et al. showed that sodium decatungstate (NaDT) as a catalyst together with hydrogen peroxide could be used to realize remote C–H oxidation of aliphatic amines under acidic conditions (Figure 4a,b).^29^ Protonation of the amine renders the α-C–H bond less susceptible to HAT, favoring distal positions. Piperidine-derived **2a** features more than one distal reactive site, and regioisomer mixtures resulted, as expected (Figure 4c). Surprisingly, when azepane-derived **2b** was used, the reaction proceeded with exclusive site-selectivity for the γ-C–H bond, despite also possessing two distal sites. This divergence led the authors to tentatively suggest that the origin may be due to an ion-pairing interaction between the protonated nitrogen of the azepane and the decatungstate anion, directing the HAT step to the γ-C–H bond and bypassing the β. Although speculative, this intriguing observation hints at the possibilities of designing dedicated systems using ion-pairing for control of site-selectivity in NaDT photocatalysis.

**Figure 5:**
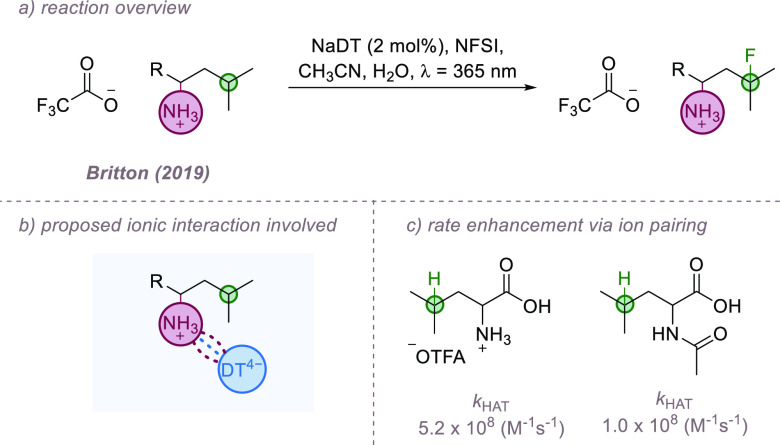
Fluorination of amine salts, accelerated by a putative ion-pairing interaction.

In an interesting report also employing NaDT-catalyzed HAT, Britton and co-workers provided convincing evidence that ion-pairing interactions between the anionic decatungstate catalyst and protonated amine substrate enhanced the rate of C–H abstraction at a tertiary position (Figure 5a,b).^30^ The resulting alkyl radical was then trapped using *N*-fluorobenzenesulfonimide (NFSI) as a fluorine atom source and demonstrated on a variety of amino acids and pseudopeptides. Comparison of reaction rates suggested that HAT occurred around five times faster on a protonated amine substrate in comparison to an analogous neutral, *N*-acetylated analogue (Figure 5c). In addition, the authors found that cationic ammonium groups incorporated elsewhere in the molecule gave rise to similar accelerating effects. While the site-selectivity was not influenced by the proposed interaction in this case, this example is notable in the context of this Perspective as it illustrates the clear potential for the ion-pairing strategy to be extended to selectivity control in a suitable substrate.

**Figure 6:**
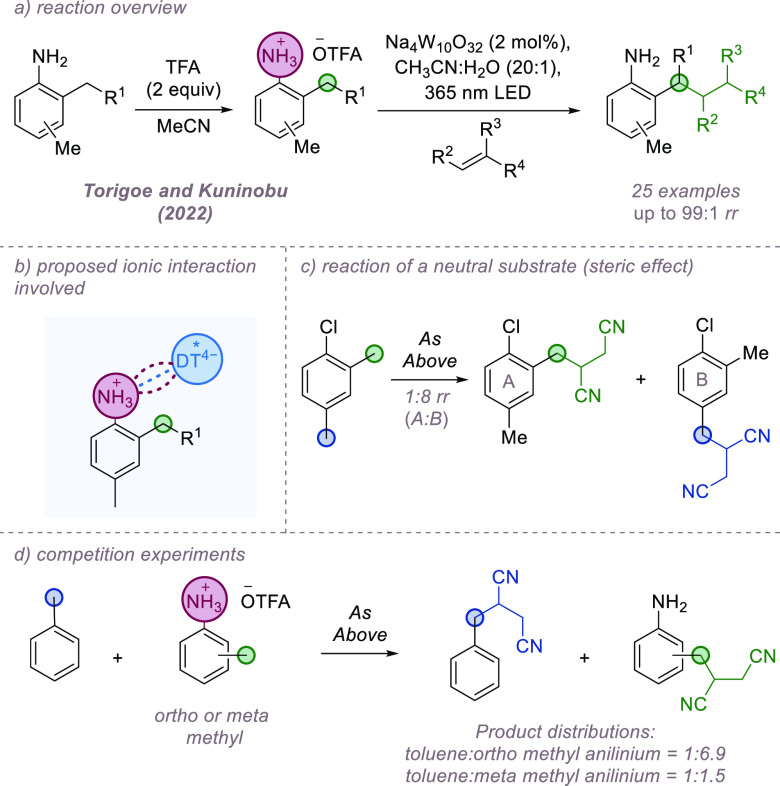
Site-selective alkylation of protonated anilines bearing methyl groups at different positions.

Developing this theme further, a recent report by Zeng, Torigoe, and Kuninobu demonstrated proof-of-concept that ion-pairing can enable site-selective HAT using decatungstate photocatalysis.^31^ Upon photoexcitation, NaDT was used to abstract a hydrogen atom from the benzylic position of anilines that possess competing methyl groups as arene substituents, with the resulting benzylic radical trapped by an electron-deficient alkene (Figure 6a). The reaction proceeded with very high site-selectivity for the methyl substituent at the aniline *ortho* position. The authors propose that the substrate is initially protonated by TFA forming the anilinium trifluoroacetate salt. An ion-pairing interaction can then be established between the cationic anilinium ion and the anionic decatungstate catalyst, promoting site-selective HAT at the proximal methyl group. For comparison, neutral 2,4-dimethylchlorobenzene favored alkylation at the *para* methyl group, suggesting the positively charged anilinium group was essential for the observed regioselectivity (Figure 6b). Alkylation of an *ortho*-methylanilinium salt was significantly preferred in an intermolecular competition experiment with toluene, despite the expected lower reactivity of the more electron deficient substrate (Figure 6c). Tellingly, the same effect was not observed with the *meta*-methylanilinium salt, providing support for the importance of proximity to the cationic nitrogen (Figure 6d).

**Figure 7:**
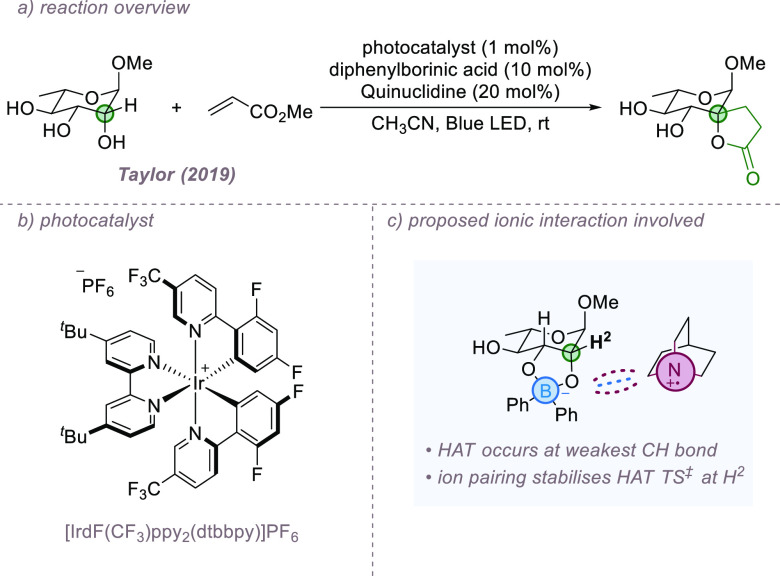
Site-selective alkylation of pyranosides in which ion-pairing interactions have been implicated.

Noncovalent interactions have been utilized extensively for addressing selectivity challenges in the synthesis and functionalization of carbohydrates.^32^ An interesting example where ion-pairing interactions have been implicated in control of site-selectivity in a C–H bond functionalization process was reported by Taylor and co-workers in 2019.^33^ Here, pyranosides were combined with catalytic amounts of diphenylborinic acid forming an anionic tetracoordinate borinic ester *in situ*. Subsequent HAT by a quinuclidinium radical cation generated via photoredox catalysis gives a carbon-centered radical, which was trapped by methyl acrylate, generating a spirocyclic lactone upon ring closure (Figure 7a,b). Despite a range of plausibly abstractable hydrogen atoms, very high site-selectivity was observed for a single position. While DFT analysis suggested that the observed site-selectivity is consistent with the calculated C–H BDEs in the borinate ester intermediate, the differences were relatively small. Interestingly the calculations showed a significantly lower activation barrier for the abstraction of H-2 in comparison to other available hydrogen atoms. The authors propose that this kinetic preference is due to an ion-pairing interaction between the anionic borinate ester and quinuclidinium radical cation at the transition state (Figure 7c).

**Figure 8:**
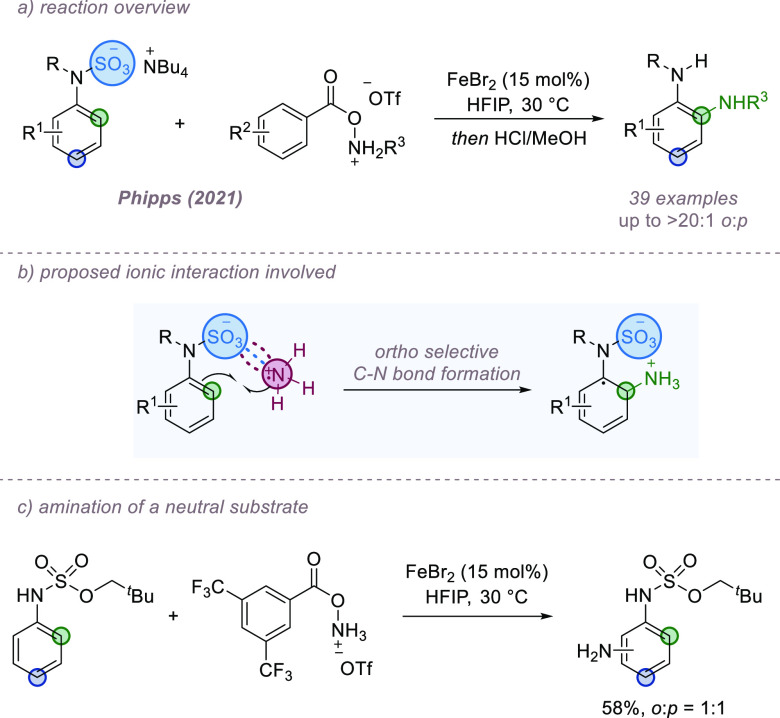
*ortho*-Selective amination of aniline-derived sulfamate salts.

Moving away from HAT-driven functionalization of C(sp^3^)–H bonds, our own group has recently developed an ion-pairing strategy to control site-selectivity in the radical functionalization of arene C–H bonds (Figure 8a).^34^ Site-selectivity is a challenge in radical-based arene amination; multiple regioisomers are commonly obtained if substituted arenes are used. We hypothesized that if an anionic functional group, such as a readily removable sulfamate, was incorporated onto the substrate, then this may ion-pair with an incoming aminium radical cation resulting in selective reaction at the proximal *ortho* position (Figure 8b). The aminium radical cation was generated using iron catalysis from an *O*-acyl hydroxylamine reagent. Both NH_2_ and NHMe groups could be transferred selectively to a range of aniline substrates, and control experiments showed that amination of related but neutral substrates resulted in a poor regiochemical outcome (Figure 8c). Furthermore, gradually increasing the dielectric constant of the reaction solvent by the addition of water resulted in a steady reduction in observed regioselectivity, presumably due to disruption of the noncovalent interactions between substrate and radical. Hydrogen bonding is likely contributing to the observed selectivity in addition, but it seems highly likely that ion-pairing is also playing a significant role.

**Figure 9:**
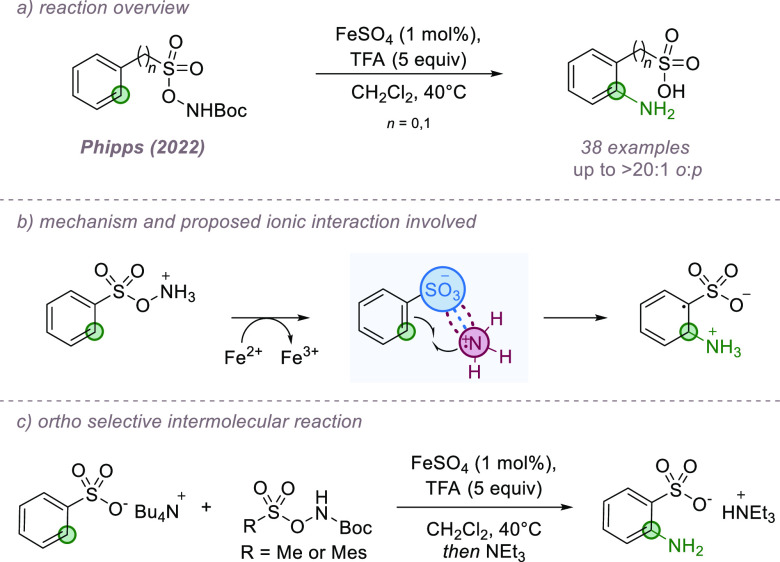
*ortho*-Selective aminative rearrangement of *O*-(arenesulfonyl)hydroxylamines.

Our group has most recently demonstrated that this concept can enable an *ortho*-selective aminative rearrangement of *O*-(arenesulfonyl)hydroxylamines.^35^ This was discovered during optimization of the earlier process and allows the facile synthesis of a diverse range of *ortho*-sulfonyl anilines (Figure 9a, *n* = 0). The addition of a methylene unit between the arene and sulfonate group was also well tolerated to give *ortho*-amino benzyl sulfonate products (Figure 9a, *n* = 1). A crossover experiment suggested that the reaction most likely proceeds via an intermolecular mechanism. Reductive cleavage of the weak N–O bond forms a benzenesulfonate anion and an aminium radical cation, and this can be assisted by iron catalysis. These two partners interact through a combination of ion-pairing and hydrogen bonding interactions, allowing selective reaction at the *ortho* position (Figure 9b). In support of this, we found that reacting tetrabutylammonium benzenesulfonate with an external source of the aminium radical cation resulted in a fully *ortho* selective amination, providing further support for an intermolecular mechanism and ion-pairing interactions being crucial for the observed regioselectivity (Figure 9c).

### 2.2 Enantioselectivity

The control of enantioselectivity in radical reactions has long represented a challenge^36^ but has received increased attention in recent years due to the popularization of photoredox catalysis.^37^ In addition to an array of important advances using covalent organocatalysis,^38^ there has been increasing attention in applying noncovalent approaches, an area that has been reviewed recently.^39^ In this Perspective, the focus is on processes that constitute C–H bond functionalization and in which ion-pairing is thought to play a crucial role in the outcome. As mentioned earlier, we will not cover carbonyl functionalization but at this point draw the interested reader’s attention to the work of Melchiorre and co-workers who developed a radical perfluoroalkylation of β-ketoesters under phase-transfer conditions using a chiral cation.^40^

**Figure 10:**
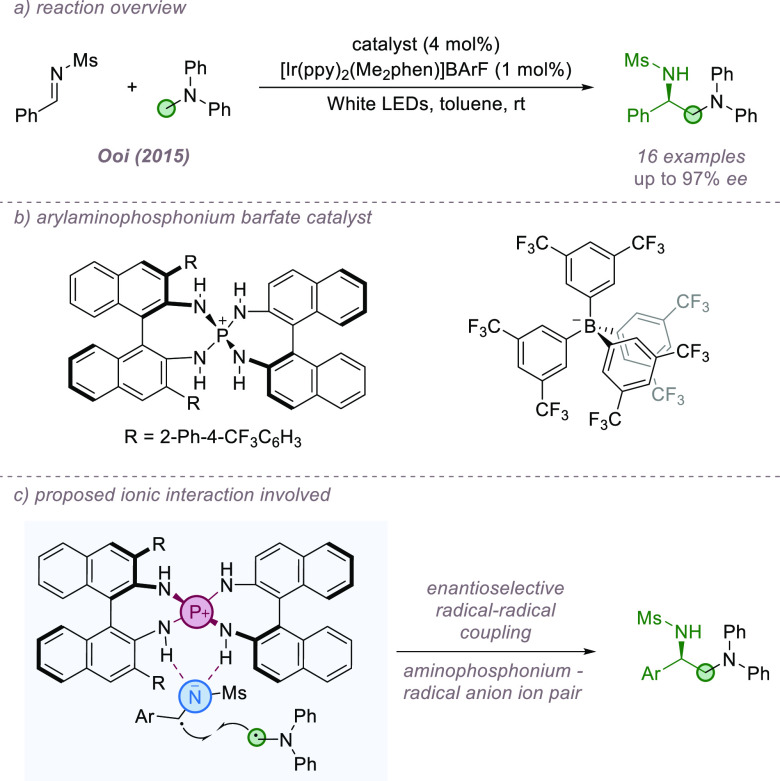
Asymmetric coupling of *N*-sulfonyl imines and alkylamines via a radical mechanism using a cationic catalyst.

An important example was reported in 2015 by Ooi and co-workers in a radical–radical coupling in which one of the partners is anionic and engages in ion-pairing interactions with a chiral cationic catalyst (Figure 10a,b).^41^ For the neutral partner, a carbon centered radical is formed through single electron amine oxidation followed by deprotonation, constituting a formal C–H bond functionalization. Concurrently, an *N*-sulfonyl imine can undergo single electron reduction to form a persistent radical anion, mediated by an iridium photoredox catalyst. It is proposed that the radical ion forms an ion pair with a chiral arylaminophosphonium cation, also a proficient hydrogen bond donor, which allows the subsequent radical–radical coupling to occur with high levels of enantioselectivity under catalyst control (Figure 10c). A particularly appealing aspect of this reaction is that it is redox neutral, and the high degree of selectivity achieved provides optimism that similar strategies could be used for enabling selectivity control in other reactions of radical anions, of which a number exist.

A related example, from Hepburn and Melchiorre, concerns the addition of oxidatively generated α-amino radicals to vinylpyridines, the latter activated using Brønsted acid catalysis.^42^ The majority of examples in this report utilized achiral acids but a single example using 3,3′-bis(2,4,6-triisopropylphenyl)-1,1′-bi-2-naphthol cyclic monophosphate (TRIP) was shown to give an encouraging 35% ee.

**Figure 11:**
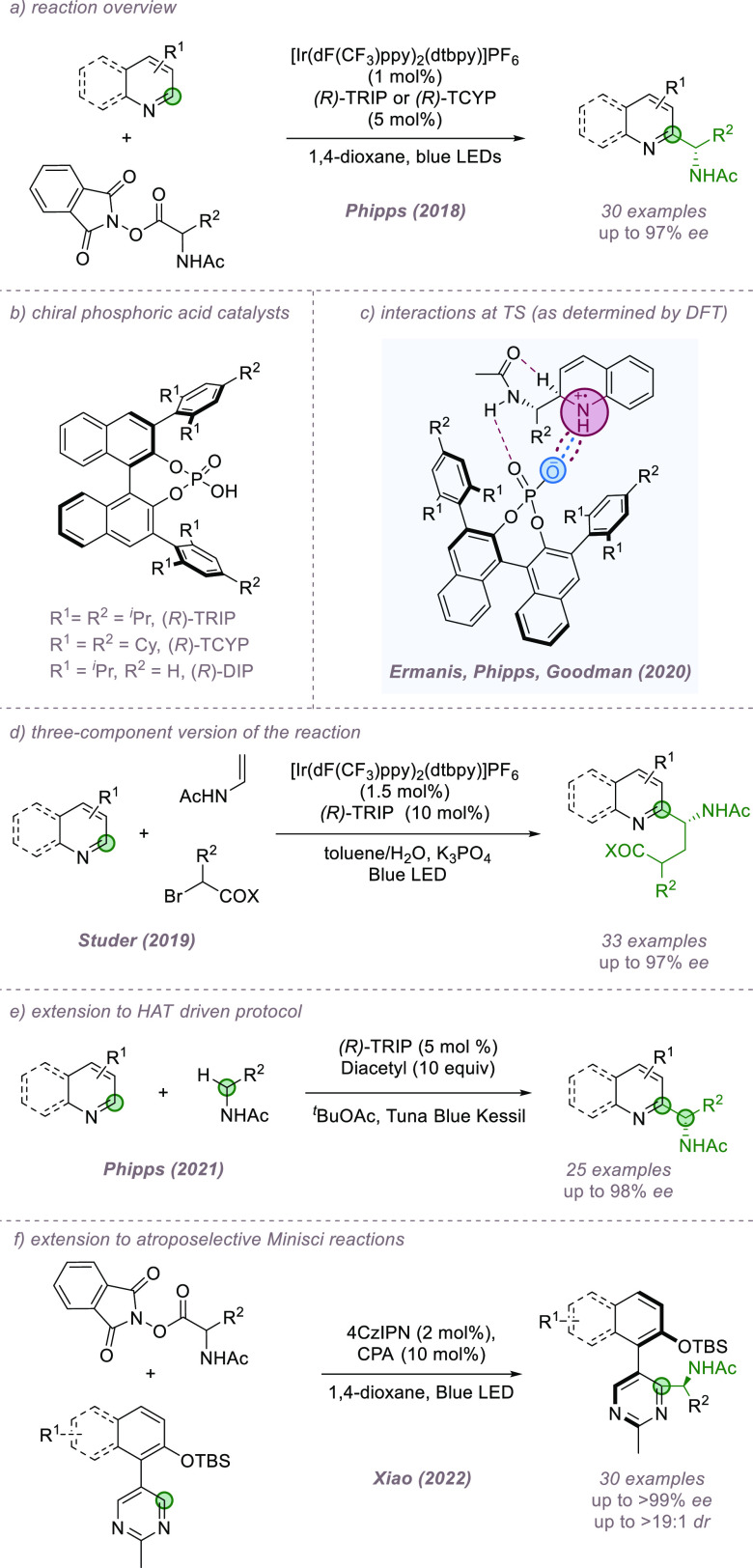
Enantioselective Minisci addition of α-amino radicals to basic heteroarenes, featuring key ion-paired intermediate. CPA: Chiral Phosphoric Acid.

The Minisci reaction constitutes a formal C–H functionalization of basic heteroarenes and follows a radical mechanism.^43^ Our group has been active in the development of catalyst-controlled Minisci reactions, which enable control over enantioselectivity if prochiral radicals are used, as well as site-selectivity at the heteroarene. In the first instance, redox-active esters (RAEs) were used in combination with iridium photocatalysis to generate pro-chiral *N*-acyl, α-amino radicals.^44^ Crucially, we discovered that the use of the chiral phosphoric acid TRIP as the catalyst allowed >20:1 site-selectivity for functionalization at the heteroarene C2 position as well as excellent enantioselectivity in the newly formed stereocenter (Figure 11a,b).^45^ The reaction was demonstrated on a range of quinolines and electron-deficient pyridines, and it was speculated, based on precedent and kinetic isotope effect (KIE) experiments, that the selectivity determining step was not radical addition but deprotonation of the subsequently formed radical cation intermediate by its associated chiral phosphate anion. The precise interactions involved in this deprotonation step were subsequently probed by the authors in collaboration with Ermanis and Goodman in a detailed experimental and computational study.^46^ After exploring a series of plausible deprotonation modes, DFT analysis revealed the lowest energy mode to be an internal deprotonation of the radical cation, carried out by the amide group with the assistance of the associated chiral phosphate, which is closely ion-paired with the intermediate (Figure 11c). Since the publication of our first protocol, a number of further developments have been made, in all cases using α-amino radical nucleophiles. This includes early work by Jiang and co-workers on a modified catalyst combination that allowed good results to be obtained with isoquinolines.^47^ In collaboration with Sigman, we developed and applied a predictive model for the reaction that guided successful expansion of the scope to diazines.^48^ Zheng and Studer developed a three-component version of the reaction where the *N*-acyl, α-amino radical is assembled from an α-bromo ester and enamide (Figure 11d).^49^ We subsequently modified our original protocol to dispense with the RAE; the requisite α-amino radicals could be formed by HAT, constituting an overall double C–H functionalization process (Figure 11e).^50^ Very recently, Xiao and co-workers published a method for the construction of heterobiaryls bearing both axial and central chirality using the enantioselective Minisci protocol (Figure 11f).^51^ In all of these cases, ion-pairing between the radical cation intermediate and the chiral phosphate anion is likely to be crucial and underpins the outstanding breath and selectivity achieved in this class of reactions.

**Figure 12:**
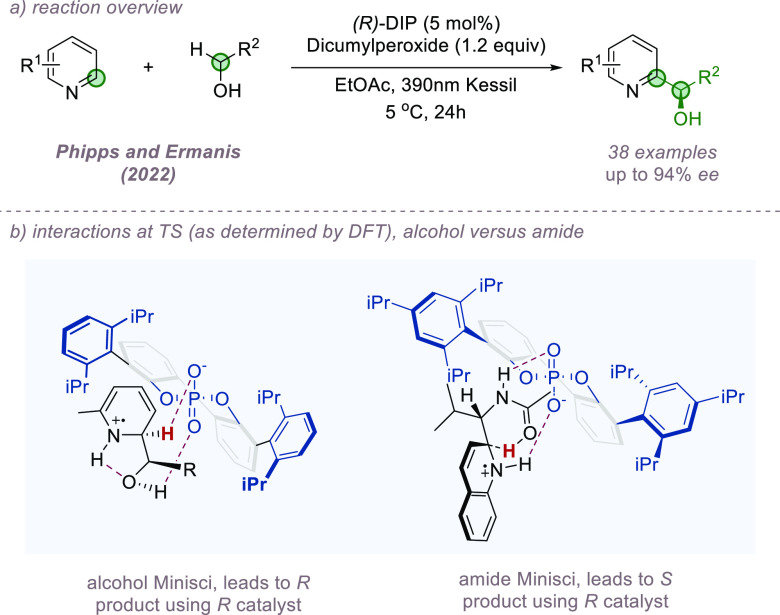
Enantioselective Minisci addition using α-hydroxy radicals proceeding via an ion-paired intermediate.

All examples of enantioselective Minisci reactions to that point had utilized α-amino radicals as nucleophiles, and the importance of this motif was highlighted in DFT analysis of the deprotonation mode. Seeking to address this limitation, we recently demonstrated that enantioselective Minisci reactions using α-hydroxy radicals are also viable. This was achieved using a HAT-driven approach and again constitutes a double C–H bond functionalization process.^52^ A range of primary alcohols could be coupled with variously substituted pyridines with excellent control of site-selectivity for the pyridine C2 position and good to excellent enantiocontrol, although with some reduction compared with the analogous amide version (Figure 12a). As before, the ion-pair comprising the chiral phosphate and the radical cation adduct following radical addition is the key complex in determining the stereochemical outcome. DFT analysis revealed that the mode of deprotonation is, as expected, quite distinct from the amide substrates. In the absence of an amide, the associated phosphate anion performs the deprotonation itself, and the lowest energy mode involves two hydrogen bonds: one between the phosphoryl oxygen and the alcohol and another between the hydroxy oxygen and the NH of the radical cation. This model predicts that the same catalyst enantiomer should lead to the opposite product enantiomer to that which was obtained in the amide Minisci and indeed this was observed experimentally (Figure 12b).

**Figure 13:**
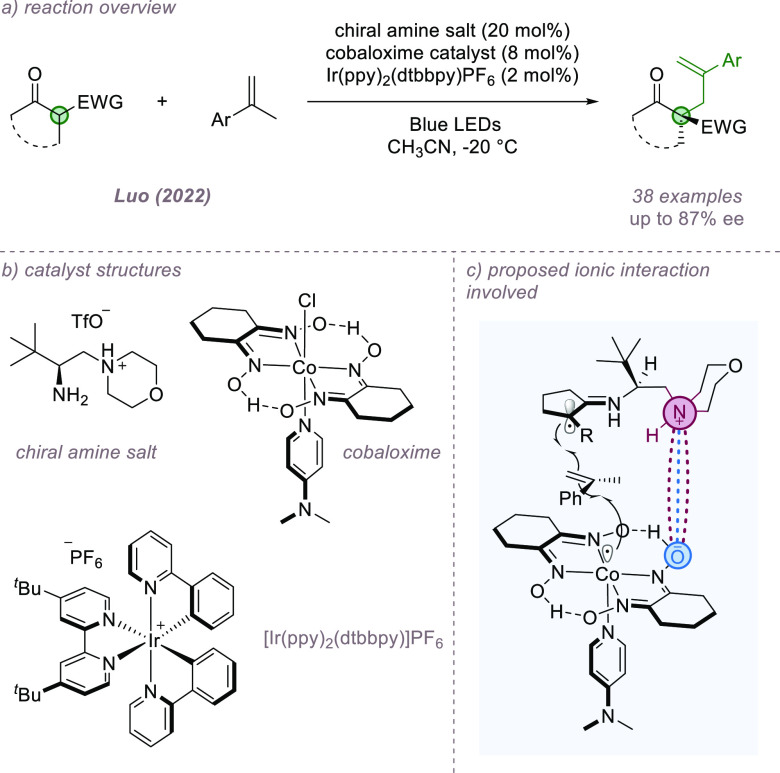
Asymmetric dehydrogenative allylic alkylation of β-ketoesters in which ion-pairing is implicated.

Luo and co-workers recently reported an enantioselective dehydrogenative allylic alkylation of β-ketocarbonyls utilizing a ternary catalytic system involving a chiral primary amine bearing a protonated morpholine unit, a photoredox catalyst, and a cobaloxime cocatalyst (Figure 13a,b).^53^ Although a carbonyl functionalization, it constitutes a C–H bond functionalization on the alkene component. The authors propose a mechanism whereby, after chiral enamine formation, the iridium photocatalyst oxidizes the enamine to give a prochiral carbon-centered radical. This and the Co(II)-metalloradical then add cooperatively to the styrene giving an alkyl cobalt intermediate, which, upon loss of H_2_, forms the product with high enantioselectivity. The authors propose a crucial ion-pairing interaction between the protonated amine and anionic cobaloxime catalyst, which further stabilizes the enantio-determining transition state (Figure 13c). In support, the authors observed a detrimental effect on enantioselectivity when more polar solvents were used.

In summary, use of ion-pairing interactions to control site-selectivity in radical-based C–H transformations is an emerging area with great potential. Given the increasing use of HAT to initiate radical reactions and the obvious challenges associated with site-selectivity, it can only be expected that interest in this approach will increase. Proof-of-concept studies have already emerged demonstrating that ion-pairing can be an effective tool, and it can be expected that these should now spur other researchers to try to apply similar strategies. In terms of enantioselective reactions falling into this category, there are relatively few so far, but considering the power of HAT, it would be surprising if further enantioselective, desymmetrizing HAT processes were not soon developed.

## 3 Ionic Mechanisms

### 3.1 Site-Selectivity

**Figure 14:**
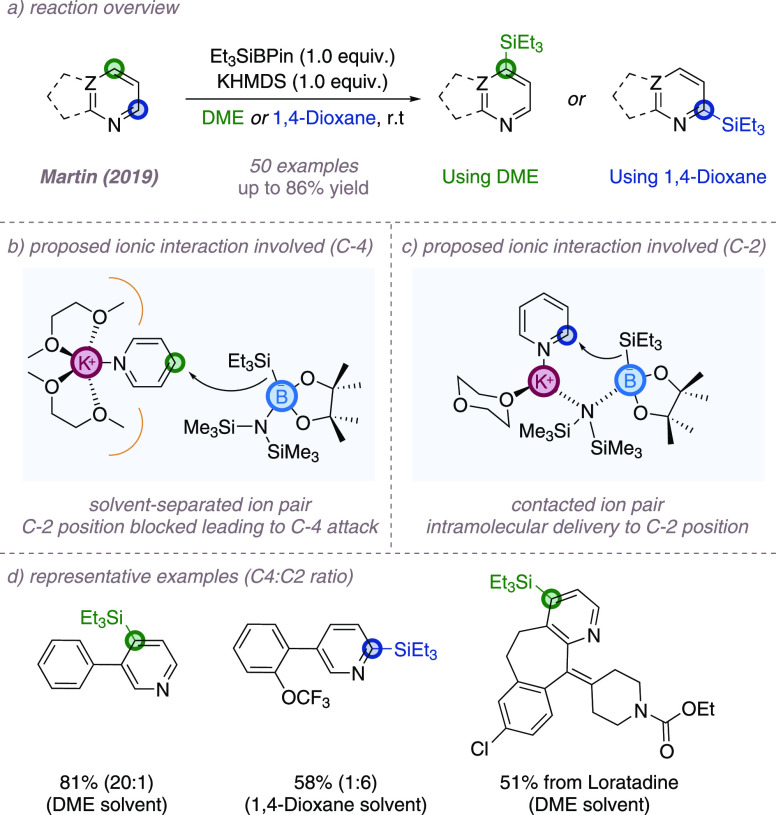
Site-selective C(sp^2^)–H silylation of (poly)azines enabling access to functionalization at either C2 or C4.

In this section, we highlight several recent examples in which ion-pairing interactions are thought to be key in the control of site-selectivity in azine functionalization via ionic mechanisms. In the first, Martin and co-workers reported a regioselective silylation reaction of azines using silyl anions to yield products containing a silicon handle for further functionalization (Figure 14a).^54^ Crucially, the methodology can introduce the silyl group with good control for either the C-2 or C-4 position, complementing established silylation methods for electron-poor azines, which typically favor C-3. After optimization, reaction of the (poly)azine with a single equivalent of both potassium bis(trimethylsilyl)amide (KHMDS) and Et_3_SiBPin (BPin = bis(pinacolato)diboron) afforded the silylated products in good yields and with good selectivity. Careful choice of solvent holds the key to the site-selectivity. When performed in dimethoxyethane (DME), the basic azine nitrogen atom is proposed to complex to a potassium ion, which is in turn complexed by two molecules of bidentate solvent. These complexation effects are thought to activate the azine to nucleophilic attack, spatially separate the potassium cation and the silyl anion, and also block off the C-2 position from the bulky nucleophile. As a result, silylation occurs at the activated C-4 position instead (Figure 14b). In contrast, in monodentate solvents such as 1,4-dioxane, C-2 selectivity is obtained. Here the cation is not fully complexed and a contact ion pair forms, which can guide the silylating agent intramolecularly to the proximal C-2 (Figure 14c). Additionally the authors demonstrated that complete C-2 selectivity could be achieved through the use of preactivated azine *N*-oxides, and the reaction was demonstrated on various drug molecules (Figure 14d).

**Figure 15:**
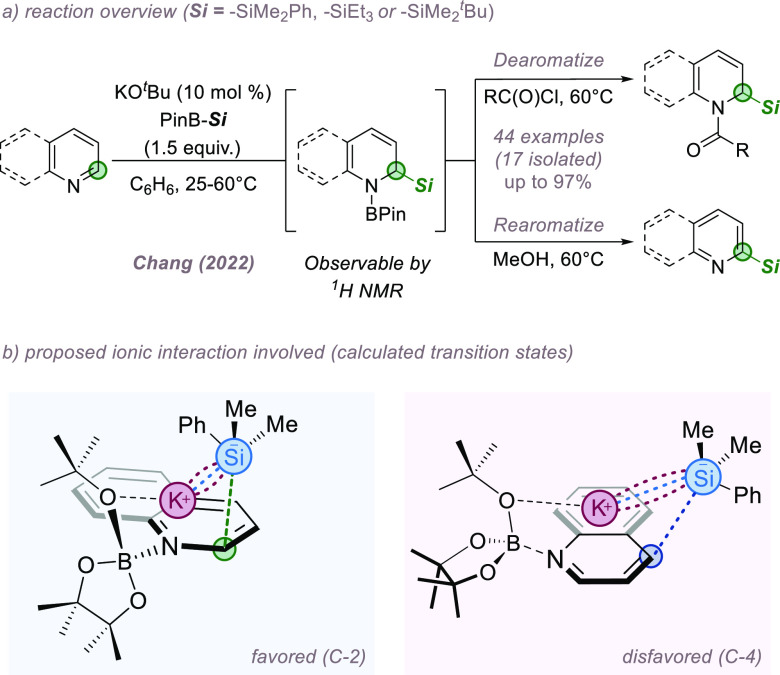
C-2 selective C(sp^2^)–H silylation of azines.

In 2022, Chang and co-workers reported a 1,2-silaboration of *N*-heteroarenes to afford either dearomatized or rearomatized C-2 silylated products (Figure 15a).^55^ Optimization established the importance of catalytic KO^*t*^Bu, which activates both the basic heterocycle (through the Lewis-acidic K^+^ cation) and the silylborane reagent (through association of ^*t*^BuO^–^ with the boron atom). The optimization also revealed differing reactivity upon variation of the silyl anion source. While the partially reduced silaborated products formed could be studied by ^1^H NMR, they proved troublesome to isolate so the authors devised telescoped protocols in which the initial adducts could be either acylated to afford the dearomatized allyl amide/cyclic enamide derivatives or alternatively rearomatized to afford the C-2 silylated analogues of the parent heterocycles. DFT revealed that in the transition state leading to C-2 silylation the LUMO of the azine is lowered in energy through coordination to boron while the potassium cation acts as an organizing element, simultaneously interacting with the boron complex and the silyl anion formed *in situ*. This results in a tight, six-membered chair transition state assembled around the potassium cation, which effectively guides the silyl anion to the C-2 position (Figure 15b). In contrast, the competing transition state leading to C-4 attack is disfavored by over 5 kcal/mol.

### 3.2 Enantioselectivity

Most examples of ionic, enantioselective C–H bond functionalization reactions that involve ion-pairing feature either chiral Brønsted acid or anion binding catalysis. In many cases, a reactive, cationic intermediate is trapped by an electron-rich arene or heteroarene in a Friedel–Crafts-type process, which forms a new bond and formally constitutes a C–H bond functionalization. The key to controlling enantioselectivity is the ion-pairing of that cationic intermediate with a chiral anion or a chiral-catalyst-bound achiral anion, although in some cases hydrogen bonding will also play an important role. While there are now quite a number of reports of such transformations, these will not be exhaustively listed; rather only illustrative examples will be given for each type.

**Figure 16:**
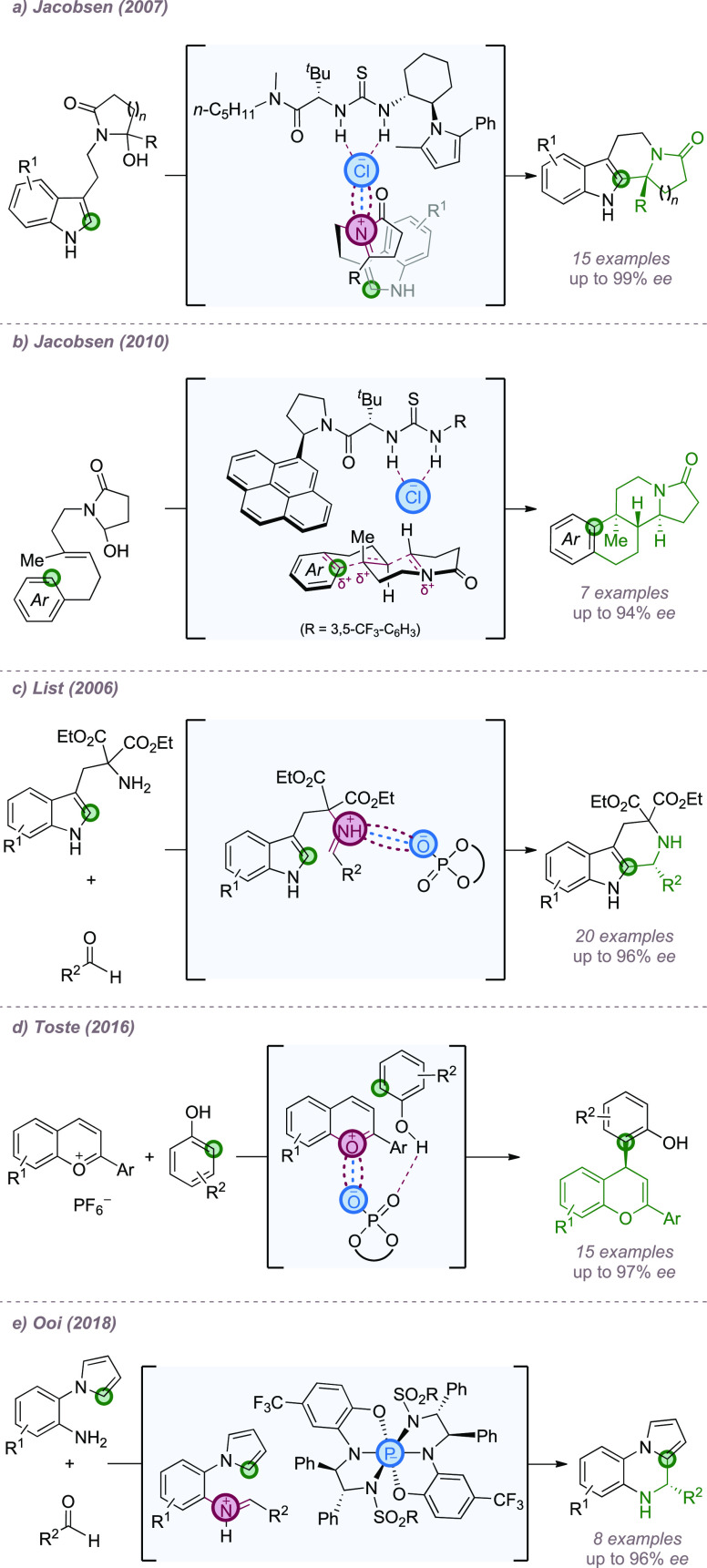
Examples of enantioselective Friedel–Crafts-type processes that exploit ion-pairing in cationic intermediates.

Chiral thiourea catalysis was investigated from an early stage. In 2004, Taylor and Jacobsen reported an asymmetric Pictet–Spengler reaction catalyzed by chiral thioureas.^56^ Subsequent detailed investigations into a closely related reaction determined that the role of the catalyst was to bind the chloride anion, which is ion paired with the *N*-acyl iminium ion intermediate, allowing the chiral catalyst to interact with this in the enantio-determining step (Figure 16a).^18a^ This insightful discovery paved the way for other related transformations, such as iso-Pictet–Spengler reactions,^57^ variants involving pyrroles,^58^ and intermolecular addition of indoles to *N*-acyliminium ions.^59^ Another elegant example from the same group involved arenes trapping a cationic intermediate in an enantioselective polycyclization reaction.^18b^ In this case, an extended aromatic substituent on the catalyst gave the highest enantioselectivies. Careful experiments lent support to the hypothesis that cation−π interactions as well as anion-binding were important in transition state organization (Figure 16b).

Chiral phosphoric acids were also used in catalytic enantioselective Pictet–Spengler reactions that involved intermediates such as protonated imines^60^ or *N*-acyliminium ions (Figure 16c).^61^ They have subsequently been used in other Friedel–Crafts type processes, an example being the asymmetric addition of phenol nucleophiles to benzopyrylium salts (Figure 16d).^62^ In all cases, the chiral phosphate is proposed to ion-pair with the cationic intermediate and control the addition of the (hetero)arene. In the cases described above, it is likely that the phosphoryl oxygen may interact with the hydrogen bond donor functionality on the nucleophile to provide a high degree of organization. In addition, in some cases, such as the example depicted in Figure 16c, it is important to consider that there is also likely to be a degree of hydrogen bonding in the interaction between the phosphate and the pronated imine. In 2018, Ooi and co-workers showcased a novel chiral hexacoordinated phosphate ion, introduced in Brønsted acid form, to catalyze a Pictet–Spengler reaction involving the C–H functionalization of pyrroles (Figure 16e).^63^ The chiral anion possessed an octahedral P(V) core consisting of two N,N,O-tridentate chiral backbones, derived from chiral diamines, and constitutes a valuable new addition to the toolkit of chiral Brønsted acids.

**Figure 17:**
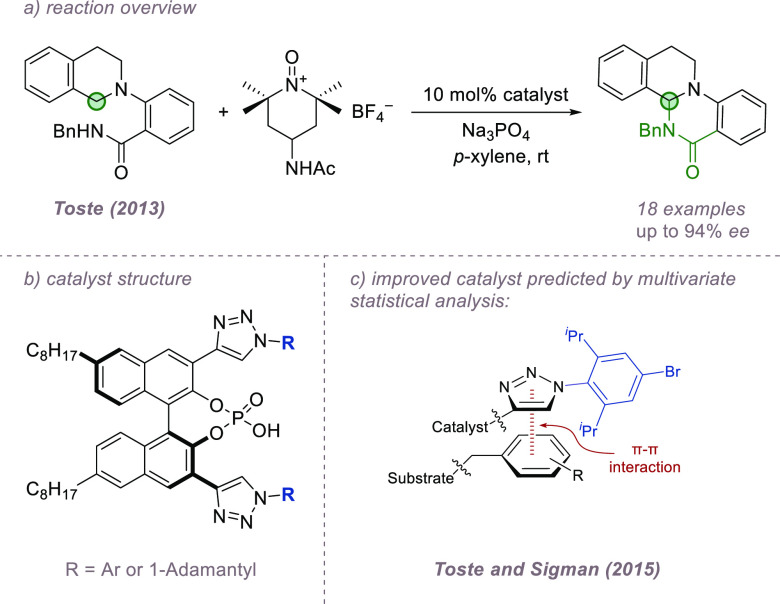
Enantioselective amination of tetrahydroisoquinolines using triazole-containing phosphoric acids.

Tetrahydroisoquinolines are a particular class of amines that are relatively easy to oxidize to imines or iminium ions. If trapped by nucleophiles, this formally constitutes a C–H functionalization process if both steps occur in the same reaction. Several such reactions invoke ion-pairing between a chiral anion and iminium intermediate to control asymmetry, and some important examples will be discussed here. In 2013, Toste and co-workers explored structurally distinct chiral phosphoric acids in which the bulky 3- and 3′-substituents were triazoles to achieve intramolecular trapping with an amine (Figure 17a,b).^64^ The chiral phosphate was proposed to act as an anionic phase-transfer catalyst to solubilize a cationic oxidant, resulting in the phosphate becoming associated with the iminium ion intermediate after oxidation. Intriguingly, the triazole-based catalysts gave opposite and enhanced enantioselectivities relative to more conventional chiral phosphoric acids, and the authors proposed that the triazole arms of the catalyst may be engaging in attractive noncovalent interactions with the substrate in addition to the ion-pairing interaction. This hypothesis was explored in a subsequent influential study in collaboration with Sigman, which used a data-intensive approach to deriving and then predictively applying a mechanistic model.^65^ This mechanistic model supported the idea that the triazole was engaging in an attractive π–π interaction with the substrate benzyl substituent at the transition state (Figure 17c). The developed model suggested that the torsion angle between the triazole ring and its *N*-substituent was a crucial parameter, and this insight allowed further rational optimization of the catalyst structure.

Also concerning tetrahydroisoquinoline functionalization, Wang and co-workers used enamine catalysis to add cyclic ketones to the intermediate iminium ions generated *in situ*.^66^ Simple amino acids were used as catalysts, and the authors proposed that an ion-pairing interaction may occur between the carboxylate group on the chiral enamine and the iminium ion. Although the scope is relatively limited, this is an interesting proposal for the use of ion-pairing interactions in the context of bifunctional catalysis.

**Figure 18:**
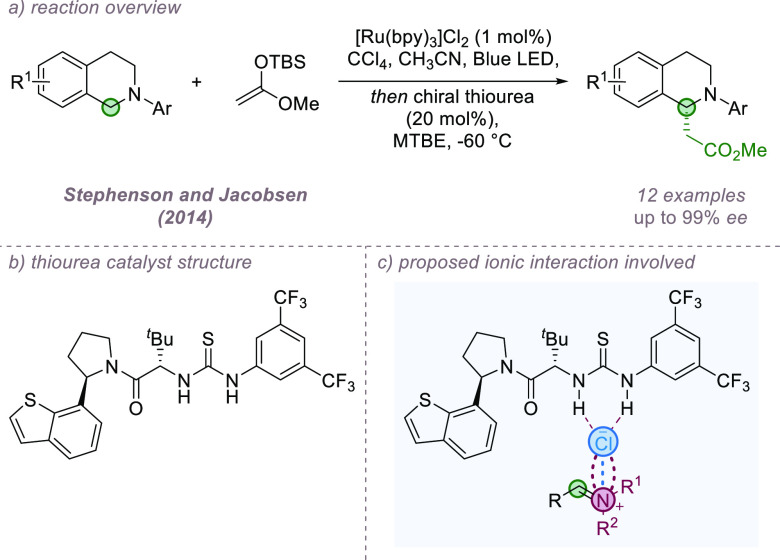
Enantioselective synthesis of β-amino esters using anion binding catalysis.

In 2014, Jacobsen, Stephenson, and co-workers reported a combination of photoredox catalysis together with anion-binding asymmetric catalysis to achieve the enantioselective functionalization of tetrahydroisoquinolines using silyl enol ethers (Figure 18a,b).^67^ Single electron oxidation of the tetrahydroisoquinoline followed by deprotonation gave an α-amino radical, which was trapped by a chloride source. Collapse of the resulting α-chloroamine gives the iminium chloride salt, and binding between the chiral thiourea catalyst and chloride anion allows enantioselective addition of the nucleophile (Figure 18c). In this case the enantio-determining step of the reaction involves ionic intermediates so it has been included here, rather than in the radical section.

In summary, the application of ion-pairing interactions to control enantioselectivity in reactions involving cationic intermediates is now fairly well-established and has been applied to several important reaction types that involve the formal functionalization of C–H bonds, as detailed here. Discrete chiral anions such as chiral phosphates have been used, as have anion-binding strategies using chiral thiourea catalysts. The charge inverted situation, which would use chiral cations, has been explored extensively in asymmetric phase transfer catalysis for enolate alkylation (not discussed here). In general, unless organic anions are stabilized, such as in enolates, then opportunities for carrying out asymmetric catalysis with them are limited. There are few examples of control of site-selectivity in this category, perhaps because of fewer available reactivity opportunities in the ionic mechanism class than there are using transition metals (see next section).

## 4 Transition Metal Catalysis

### 4.1 Site-Selectivity

Transition metal catalysis offers a wealth of reactivity opportunities. In ideas explored in this section, the primary strategy employed is to exploit an ionic interaction between part of a catalyst’s architecture and a substrate to control site-selectivity in a transition-metal-catalyzed reaction. This often involves careful design of a bifunctional ligand bearing a charged group, which can engage appropriate substrates in ionic interactions while simultaneously modulating the reactivity of the metal that it supports. In designing such ligands, one must ensure that the newly incorporated ionic portion is not detrimental to the activity or solubility of the catalyst. If successful, this approach can powerfully complement the often-remarkable reactivity of transition metal catalysts, combining optimal reactivity with tunable selectivity.^5a^

Iridium-catalyzed arene C–H borylation has become firmly established as the benchmark C–H functionalization reaction in which to test new ligand designs that exploit noncovalent interactions to influence site-selectivity.^19b,68^ In addition to providing versatile products, the transformation is unique in that the natural regioselectivity is overwhelmingly governed by the steric demands of the substrate, with electronic and proximity effects playing a secondary role.^20b^ This often leads to mixtures of regioisomers if monosubstituted or 1,2-disubstituted arenes are employed. As a result there has been much recent effort to customize ligands to enable borylation at a single arene position, with a number of these utilizing ionic interactions.

**Figure 19:**
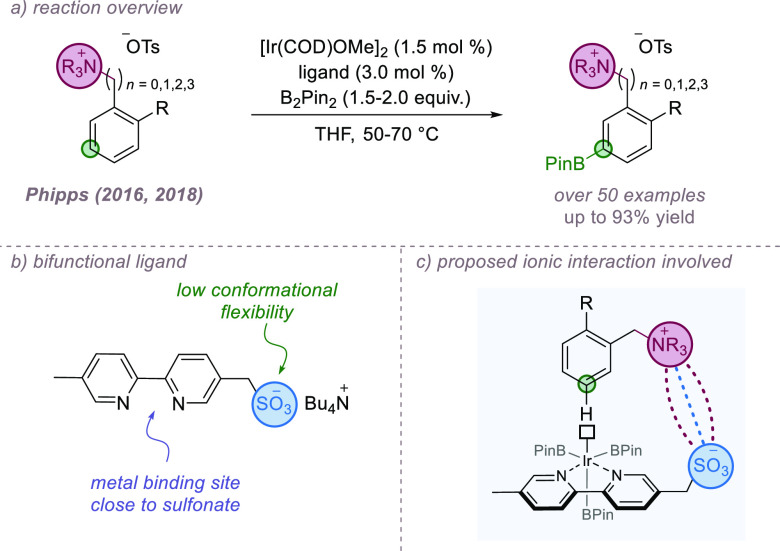
Sulfonated bipyridine ligand enables *meta*-selective arene borylation of cationic substrates.

In 2016, our own group reported the *meta*-selective borylation of aromatic quaternary ammonium salts derived from anilines and benzylamines (Figure 19a).^69^ The selectivity was achieved through an attractive ion-pairing interaction between the cationic substrates and an anionic sulfonated bipyridine ligand, capable of positioning the sterically accessible *meta* position close to the metal center (Figure 19b,c). Control experiments in which the ability to form the ionic interaction was removed or attenuated, either through the use of neutral substrates or through addition of an external cationic competitor led to dramatic losses in selectivity. In a separate study, reactions employing analogous substrates in which the trimethylammonium was replaced by a phosphonium group also gave high selectivities.^70^ In 2018, it was demonstrated that the same ligand was also competent in the borylation of the longer-chain phenethylamine and phenylpropylamine-derived ammonium salts, which possess significantly increased conformational freedom. However, if the chain was made longer, selectivity started to drop as the chain flexibility increased further.^71^ The fact that a single ligand can be effective on substrates with four different chain lengths underscores the potential advantages that a looser, ionic “flexibility-to-fit” approach can confer compared to a more directional hydrogen-bonding strategy in certain situations.

**Figure 20:**
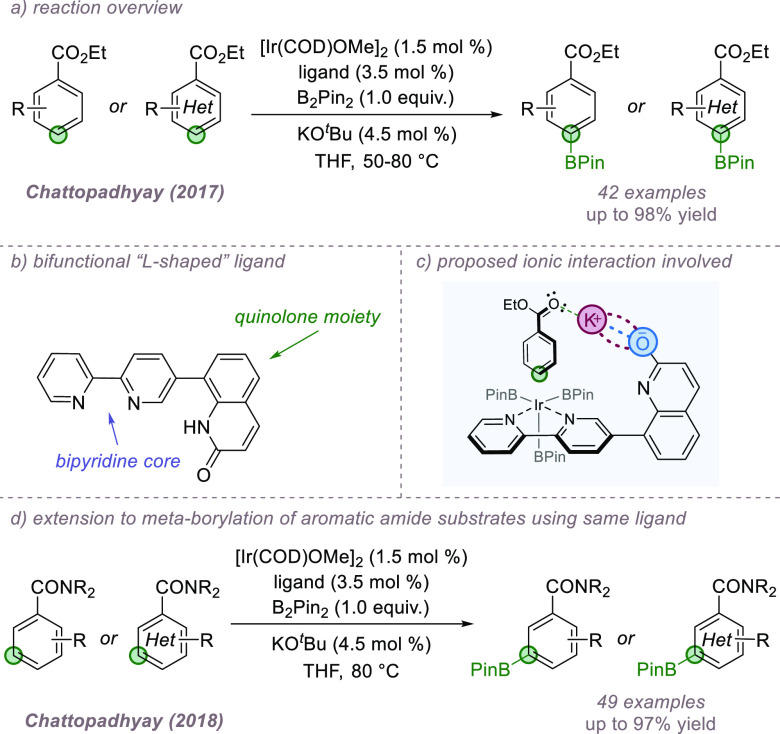
L-shaped ligand enables regioselective borylation of aromatic ester (*para*) and amide (*meta*) substrates.

Creative catalyst designs have been developed to achieve *para*-selective arene borylation, with the greater distance of this position suggesting that a more extended catalyst should be required. In 2017, the Chattopadhyay group reported a new ligand design for *para*-selective borylation of aromatic esters (Figure 20a).^72^ The ligand design unites two moieties: a bipyridine core unit for binding the iridium and a quinolone group, which can indirectly engage suitable substrates through a noncovalent interaction (Figure 20b). Under the reaction conditions, it was proposed that the 2-hydroxypyridine tautomer of the ligand is deprotonated and ion-pairs with a potassium cation. This cation engages in a cation-dipole interaction with the substrate carbonyl, orienting the *para*-position of the substrate close to the iridium center (Figure 20c). Support for the importance of the potassium cation was provided by control studies in which regioselectivity was greatly reduced when the potassium cation was either sequestered with 18-crown-6 or replaced with sodium. Intriguingly, in a subsequent report, the authors revealed that with the same ligand the selectivity of the reaction switched to the *meta* position upon changing the aromatic ester to an aromatic amide group (Figure 20d).^73^ Here, the authors speculated that the interaction between the carbonyl lone pair and the potassium ion was retained and that the selectivity was altered due to distortion from planarity of the aromatic ring, resulting in the *meta* position being closest to iridium.

**Figure 21:**
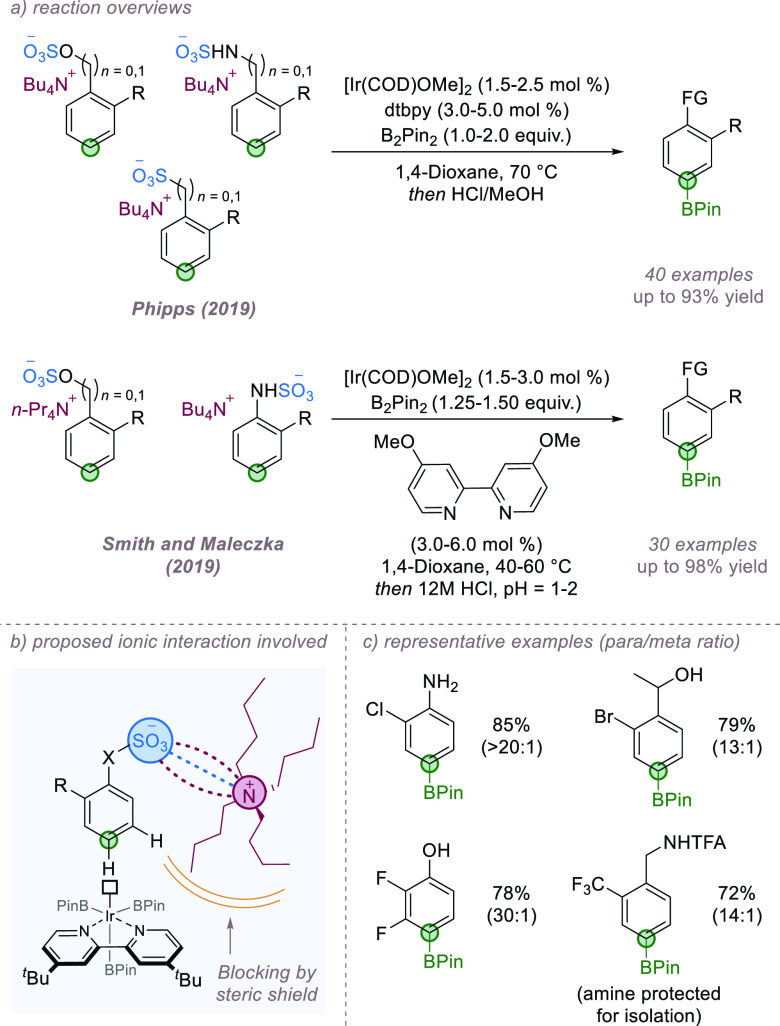
Simultaneous reports demonstrated *para*-selective borylation of common arene building blocks by virtue of an associated bulky cation.

In 2019, our group^74^ and those of Maleczka and Smith^75^ simultaneously reported a conceptually simple yet highly general *para*-selective borylation protocol of anionic substrates derived from simple arene building blocks (Figure 21a). In contrast to the examples above, the regioselectivity arose as a consequence of ion-pairing between the anionic substrate and an associated bulky cation, as opposed to with a charged catalyst. Here, standard borylation ligands were used and the reaction relied on steric-based regioselectivity. Both studies hypothesized that association of an anionic 1,2-disubstituted arene substrate with a sterically bulky tetraalkylammonium cation would selectively shield the *meta*- position and result in functionalization at the *para* position (Figure 21b). This was supported by control experiments in which an erosion of selectivity was observed upon moving to smaller cations. A variety of tetraalkylammonium sulfonate and sulfamate salts based on ubiquitous phenols, benzyl alcohols, anilines, and benzylamines all underwent selective borylation, as did aryl and benzyl sulfonates (Figure 21c). In addition to the excellent selectivities and yields, the reactions boast operational simplicity with facile protocols to both install and remove the temporary anionic groups.

**Figure 22:**
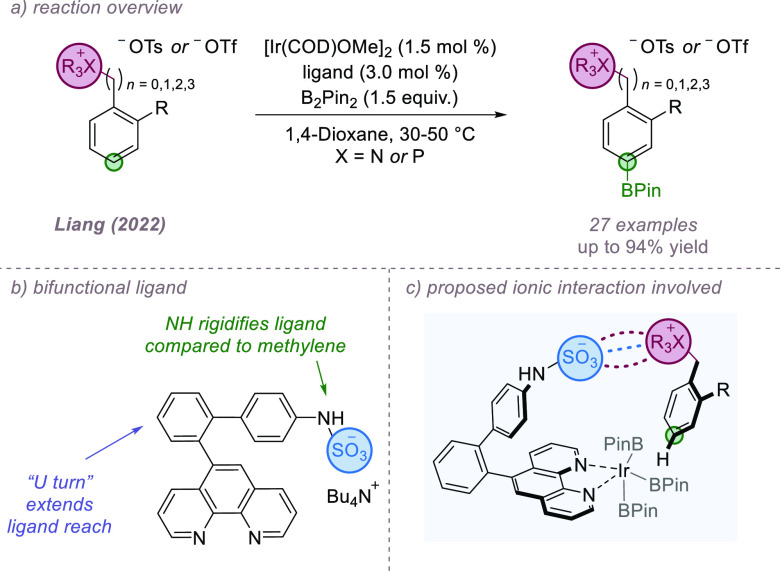
Extended biphenyl-phenanthroline sulfamate ligand allows *para*-selective borylation of ammonium salts.

Returning to the direct ligand–substrate ion-pairing interaction, Liang and co-workers recently disclosed a biphenyl-phenanthroline sulfamate ligand scaffold incorporating a “U-turn” motif, which can reach further than Phipps’ original sulfonated bipyridine thus enabling *para*-selective borylation of a range of cationic quaternary ammonium and phosphonium salts (Figure 22a,b).^76^ In addition to the greater reach of the ligand, the authors found that a nitrogen atom linker between the sulfonate group and the aromatic ring of the ligand improved reactivity and selectivity as a result of enhanced ligand rigidity. DFT calculations revealed that the electrostatic interactions operating in the transition states leading to the *meta*- and *para*-products were almost identical and were not responsible for the high selectivity (Figure 22c). Rather, unfavorable distortion of the phenanthroline moiety elevated the energy of the transition state leading to the *meta* isomer, disfavoring its formation. In a recent publication, Douthwaite and Phipps reported related but less extended ligand designs based on bipyridine, although the selectivities were generally low.^77^

### 4.2 Enantioselectivity

**Figure 23:**
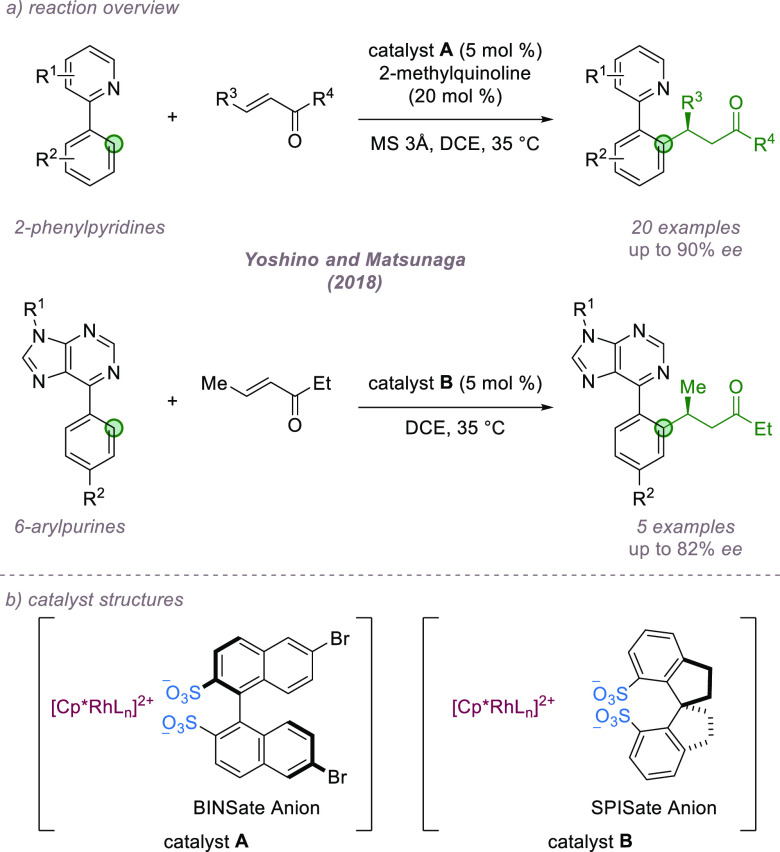
Enantioselective C–H functionalization using a chiral anion for a cationic Cp*Rh(III) complex.

As mentioned previously, the potential for chiral cations to impart enantioselectivity in organocatalytic reactions has been well appreciated through extensive research in asymmetric phase-transfer catalysis.^10c^ In a charge-inverted sense, chiral anions have been used to induce asymmetry in challenging transition-metal-catalyzed reactions, which involve cationic metal complexes.^13^ An interesting application of this strategy to C–H activation catalyzed by a chiral pentamethylcyclopentadienyl rhodium(III) catalyst was reported in 2018 by Yoshino, Matsunaga, and co-workers (Figure 23a).^78^ Induction of enantioselectivity in Cp*M(III)-catalyzed C–H functionalization can be challenging given that there are no vacant coordination sites at the metal during the C–H insertion step, a problem that has been partially addressed using creative chiral cyclopentadienyl ligand designs based on steric-blocking approaches.^79^ Here the authors hypothesized that association of an achiral *cationic* Cp*M complex with a chiral disulfonate anion might enable enantioinduction via ion-pairing. The authors reported that a Cp*Rh(III)/(*S*)-1,1′-binaphthalene-2,2′-disulfonate ((*S*)-BINSate) catalyst was effective in catalyzing the asymmetric conjugate addition of 2-phenylpyridines to α,β-unsaturated ketones (Figure 23b). Changing the chiral disulfonate from BINSate to the spirocyclic SPINSate enabled a scope expansion to include 6-arylpurines. The authors speculate that the chiral disulfonate acts either as a chiral counteranion or as a chiral proton source with further mechanistic experiments required to distinguish between these two scenarios.

**Figure 24:**
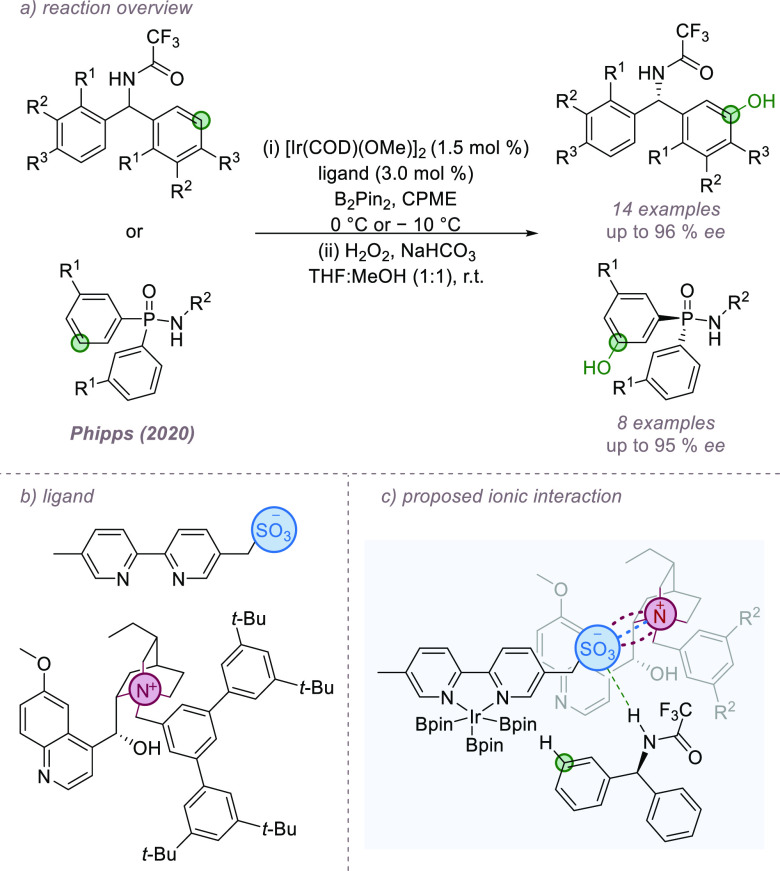
Enantioselective, desymmetrizing arene borylation directed by a chiral cation.

There are relatively few examples of combining chiral cations with transition metals, since catalytic cycles rarely contain anionic metal intermediates, precluding direct ion-pairing between a catalytically relevant complex and a chiral cation. Notable exceptions, along with other related examples, have been documented in recent reviews.^11,80^ Our group has developed an alternative strategy for uniting privileged chiral cations with transition metals to carry out enantioselective C–H bond functionalization. In this approach, a common achiral ligand scaffold is first rendered anionic through the attachment of a sulfonate group, which can ion-pair with a chiral cation. In this way, the source of chirality is held close to the transition metal center throughout the catalytic cycle and can influence the enantio-determining step, potentially through a combination of repulsive steric effects and attractive noncovalent interactions. This concept was first demonstrated in an iridium-catalyzed desymmetrizing arene borylation. Two distinct classes of substrate, benzhydrylamides and phosphinamides, were converted to the *meta*-borylated products with excellent enantioselectivities (Figure 24a).^81^ The ligand used for the transformation was based on that used previously to achieve regioselective arene *meta*-borylation but differs in that the achiral tetrabutylammonium cation is replaced with a chiral cation based on quaternized dihydroquinine (Figure 24b). Not only is the counterion now chiral but it also possesses a clustering of various functionalities, which can engage in attractive noncovalent interactions with substrates. It was hypothesized that in this complex system, the anionic sulfonate acts as an anchor, interacting simultaneously with the substrate through hydrogen bonding and with the cation through ion-pairing. This brings the key components together in a highly organized ternary complex (Figure 24c). It was found that benzhydrylamide substrates that pose a regioselectivity choice to the catalyst were borylated with excellent regioselectivity in addition to enantioselectivity.

**Figure 25:**
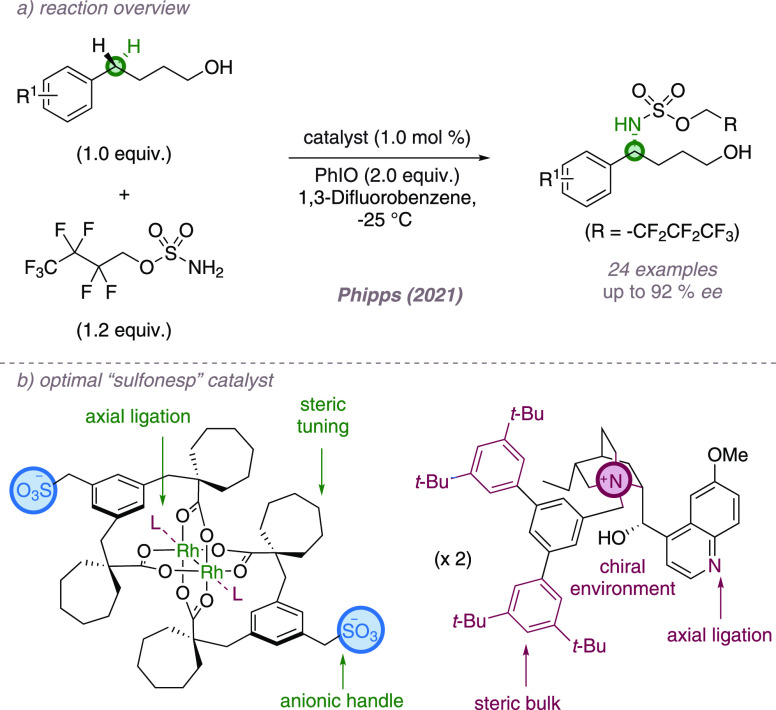
Enantioselective C–H amination, directed by a chiral cation.

We were next intrigued to investigate whether this approach could be applied to other important C–H functionalization reactions using different catalytic systems. This led us to design novel anionic dirhodium tetracarboxylate paddlewheel complexes and pair these with similar chiral cations. We wondered whether the association of a sulfonated variant of the best-in-class catalyst for intermolecular nitrene transfer, Rh_2_(esp)_2_, with a chiral cation might enable enantioselective intermolecular benzylic amination while still maintaining the excellent reactivity of the parent dimer (Figure 25a). Catalyst structure was optimized by fine-tuning both the steric profile of the achiral sulfonated ligand and the chiral cation.^82^ The substrates of choice were aryl alcohols, and we propose that the terminal hydroxyl provides a functional group that can interact with the ligand sulfonate group through hydrogen-bonding, providing organization at the transition state. In the case of simple 4-arylbutan-1-ol substrates, the optimal “sulfonesp” scaffold paired with a bulky cation derived from dihydroquinidine delivered enantioenriched 1,4-amino alcohol derivatives in good yield and good to excellent enantioselectivity (Figure 25b). Equal but opposite enantioselectivity was obtained using the desvinylquinine variant of the chiral cation. Encouraging progress toward the enantioselective amination of aryl alcohol substrates of different chain lengths using alternative sulfonated scaffold/chiral cation combinations was also presented. Given the wide-range of reactions catalyzed by Rh(II,II) dimers, we are optimistic that these dimers can be used to induce asymmetry in other transformations.

Having showcased this concept on two challenging C–H functionalization reactions with different metals and ligand systems, we anticipate that there will be further opportunities to apply it more broadly to other transition-metal-catalyzed reactions that are currently difficult to render asymmetric.

## 5 Conclusions and Outlook

In this Perspective, we have used the topical and mechanistically diverse class of reactions that can be defined by the term “C–H bond functionalization” as a lens through which to explore how ion-pairing interactions have been applied to exert control over either site-selectivity or enantioselectivity.

Site-selectivity is a challenge of pertinence to C–H bond functionalization reactions and is a deciding factor in how useful a method may ultimately be. It is clear from this survey that ion-pairing interactions are not yet mainstream but are emerging and show great promise in the situations to which they have been applied. For example, several recent studies have convincingly made the case that ion-pairing can exert control in HAT site-selectivity. HAT is increasingly recognized as a powerful and versatile tool for functionalizing molecules in ways that are very challenging using transition metals, and there is much interest in the development of HAT catalysts. Given that some HAT catalysts are themselves already charged, the opportunities here are evident. Equally, neutral HAT catalysts could be rendered ionic by attachment of appropriate groups, and we predict that this will be a fruitful avenue of research. Despite all the advances in transition-metal-catalyzed C–H functionalization, it can still be challenging to control site-selectivity, particularly when guiding the reactive transition metal to nonproximal positions to achieve remote functionalization. Ion-pairing has already risen to this challenge in the context of iridium-catalyzed borylation, a transformation increasingly used as a testbed for noncovalent directing approaches, and we anticipate that in the future this will be applied to other transition metals. For example, palladium is one of the most widely used metals for C–H functionalization, and in recent years great progress has been made in guiding it away from proximal arene positions.^20d^ One of the challenges in superimposing noncovalent strategies onto palladium-catalyzed C–H functionalization is the frequent use of high temperatures and polar solvents, which could potentially disrupt weaker interactions. As a result, the advances made using Ir-catalysis in borylation have yet to be transposed to Pd-catalysis. One might imagine that here ion-pairing interactions, exploiting Coulombic attraction, should be more resilient than hydrogen bonds and could offer unique advantages.

For control of enantioselectivity, this survey has shown that the majority of examples constituting C–H bond functionalization make use of ion pairing between (radical) cationic intermediates and either chiral anions or achiral anions bound to chiral catalysts. In the most part, these exploit the remarkably versatile BINOL-derived chiral phosphate anion or the class of thiourea-based anion binding catalysts pioneered by Jacobsen. A single example involves a radical anion intermediate paired with a chiral cation. Given that the effectiveness of chiral cations is well appreciated through their ion-pairing in asymmetric enolate alkylation chemistry, there seem to be many possibilities for pairing them with other types of radical anion. A number of distinct classes of radical anion could be classed as persistent, and this feature could make them suitable for asymmetric catalysis, an idea yet to be explored in any depth. In transition metal catalysis, enantioselective forms of C–H functionalization represent a relatively small portion of all such reactions. Reactivity is a paramount challenge; the functionalization of C(sp^3^)–H bonds using metals is still very difficult, and so the relative paucity of examples when compared with arene C–H functionalization limits situations where stereocenters may be formed. One may anticipate that, in the years ahead, further development of mild, functional group tolerant, metal-catalyzed C–H functionalization will allow it to operate in a general way on aliphatic systems. It is at this point that the ability to induce enantioselectivity will become extremely important, and it is crucial that novel concepts and methods are developed in preparation for this. To achieve mild and general C(sp^3^)–H functionalization, it is likely that the ligand environment around the metal will need to be very precisely tuned, and so asymmetric strategies will need to be carefully designed not to disrupt this. Ion-pairing catalysis may offer unique advantages in such a situation as the chirality can be located on the counterion if the metal complex is charged, either naturally or by appendage of a charge on the periphery of the complex. Proof-of-concept of such an approach has recently been demonstrated, and it is expected that this will be expanded.

Ion-pairing catalysis offers unique opportunities for selectivity control and particularly for tackling challenges that do not immediately succumb to conventional approaches. There are some misconceptions surrounding ion-pairing such as that the relatively low-directionality compared to hydrogen bonding is prohibitive for obtaining useful control or the belief that only the lowest polarity solvents can be used. We hope that the ground covered in this article can help to dispel these to some degree. Although the area as a whole is much broader than the examples covered in this Perspective, we have chosen to present the advances that intersect with a broadly defined area of chemistry that is exciting and mechanistically diverse and offers ample opportunity to innovate in order to address challenges. We hope that this will spur others to consider design strategies involving ion-pairing to exert selectivity control in all manner of methodology types in addition to C–H functionalization.
